# Mask-Transformer-Based Networks for Teeth Segmentation in Panoramic Radiographs

**DOI:** 10.3390/bioengineering10070843

**Published:** 2023-07-17

**Authors:** Mehreen Kanwal, Muhammad Mutti Ur Rehman, Muhammad Umar Farooq, Dong-Kyu Chae

**Affiliations:** 1 DeepChain AI&IT Technologies, Islamabad 45570, Pakistan; mehreen@deepchain.pk; 2 Department of Computer and Software Engineering, National University of Science and Technology, Islamabad 43701, Pakistan; mmutti.ce41ceme@student.nust.edu.pk; 3 Department of Computer Science, Hanyang University, Seoul 04763, Republic of Korea

**Keywords:** teeth segmentation, panoramic radiographs, mask-transformer-based networks, panoptic segmentation

## Abstract

Teeth segmentation plays a pivotal role in dentistry by facilitating accurate diagnoses and aiding the development of effective treatment plans. While traditional methods have primarily focused on teeth segmentation, they often fail to consider the broader oral tissue context. This paper proposes a panoptic-segmentation-based method that combines the results of instance segmentation with semantic segmentation of the background. Particularly, we introduce a novel architecture for instance teeth segmentation that leverages a dual-path transformer-based network, integrated with a panoptic quality (PQ) loss function. The model directly predicts masks and their corresponding classes, with the PQ loss function streamlining the training process. Our proposed architecture features a dual-path transformer block that facilitates bi-directional communication between the pixel path CNN and the memory path. It also contains a stacked decoder block that aggregates multi-scale features across different decoding resolutions. The transformer block integrates pixel-to-memory feedback attention, pixel-to-pixel self-attention, and memory-to-pixel and memory-to-memory self-attention mechanisms. The output heads process features to predict mask classes, while the final mask is obtained by multiplying memory path and pixel path features. When applied to the UFBA-UESC Dental Image dataset, our model exhibits a substantial improvement in segmentation performance, surpassing existing state-of-the-art techniques in terms of performance and robustness. Our research signifies an essential step forward in teeth segmentation and contributes to a deeper understanding of oral structures.

## 1. Introduction

**Figure 1:**
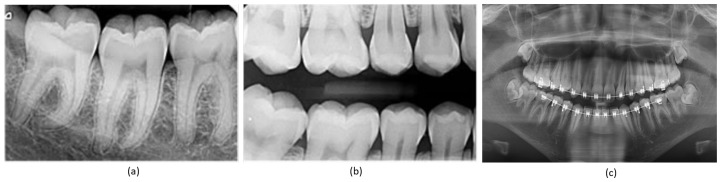
Types of X-ray images: (**a**) periapical X-ray; (**b**) bitewing X-ray; (**c**) panoramic X-ray.

Teeth segmentation is pivotal in the clinical diagnosis of oral diseases, offering essential precision in surgical planning through the accurate delineation of teeth boundaries [1,2]. In orthodontics, real-time information regarding teeth movement and root depths is crucial for immediate assessment of a patient’s dental alignment and for accelerating the orthodontic treatment cycle [3]. The prerequisite for achieving this is the precise segmentation of teeth in dental panoramic X-ray images [4], which has additional applications in forensic identification [5], age estimation, and the analysis of hidden dental structures, including benign or malignant masses [6]. Dentistry extensively utilizes radiographic images for diagnosis, given their comprehensive visualization of the internal structure of the mouth [7]. Extra-oral radiographs, encompassing panoramic and cephalometric images, capture the complete dentition and surrounding areas, providing critical insights into a patient’s teeth, as demonstrated in Figure 1. However, manual and semi-automated segmentation approaches for teeth and tissues in these radiographs often prove time consuming, tedious, and subjective, with their efficacy heavily reliant on the dentist’s expertise. Additionally, segmentation in low-quality image settings presents even greater challenges. Given these circumstances, the development of an automatic, accurate, and efficient teeth segmentation method is paramount.

Traditionally, teeth segmentation has been approached through semantic and instance segmentation techniques [8,9]. While semantic segmentation classifies each pixel into predefined classes without distinguishing between object instances, instance segmentation offers a more comprehensive understanding by segmenting objects and distinguishing each tooth object instance. Both category and instance labels are crucial in this context, which has become a focal point in dental research. However, both proposal-based and proposal-free instance segmentation approaches have their limitations. They often struggle with differentiating object instances within the same category, particularly when objects overlap, and preserving pixel-wise location information, which often results in coarse mask boundaries.

Numerous attempts have been made to develop a highly accurate automatic teeth segmentation algorithm [10,11]. However, teeth segmentation remains challenging due to fuzzy boundaries caused by low contrast and noisy dental panoramic X-ray images. The diversity of teeth conditions across different patients and the presence of dental instruments, such as metal racks and dental implants, pose significant obstacles to achieving accurate teeth segmentation. Recognizing these challenges, this research introduces a novel approach based on panoptic segmentation [12]. Panoptic segmentation unifies the typically disjoint tasks of semantic segmentation (identifying and classifying objects in an image) and instance segmentation (segmenting individual instances of each object), offering a more holistic and precise tooth and oral tissue segmentation strategy [13,14]. Several studies have shown the effectiveness of panoptic segmentation for optimizing the performance of deep-learning-based models [15,16,17,18].

We propose a panoptic-segmentation-based approach for instance teeth segmentation and surrounding tissue semantic segmentation. Panoptic segmentation, a unified framework for semantic and instance segmentation, yields better Dice scores for teeth segmentation by providing an improved context understanding, better discrimination of close or touching instances, and consistent pixel-level labeling. This approach reduces false positives and negatives by correctly segmenting teeth instances and accurately labeling non-teeth regions, enhancing the overlap between prediction and ground truth, which the Dice score measures. Our model employs a mask transformer to predict non-overlapping masks and their corresponding semantic segmentation labels directly. The panoptic quality (PQ) style loss is utilized to optimize the output masks and classes. More specifically, we design the similarity metric between consecutive teeth-labeled masks as the product of their masks and class similarity, inspired by the PQ definition. Moreover, the innovative strategies proposed by groundbreaking works that use attention mechanisms, such as [19,20], motivated us to incorporate attention modules into our proposed network.

We introduce a novel architecture to effectively train and infer using the mask transformer. Unlike traditional architectures [21,22] where the transformer is placed on top of a convolutional neural network (CNN) [23], we adopt a dual-path framework that effectively merges CNNs with transformers [24,25,26,27]. This allows CNN layers to read and write into global memory by incorporating memory-to-pixel attention (M2P), memory path self-attention (M2M), pixel–path axial self-attention (P2P), and pixel-to-memory attention (P2M). As a result, the transformer can be inserted at any position in the CNN to enable communication with the global memory at any layer. The proposed architecture also employs a stacked hourglass-style decoder [28,29] to aggregate multi-scale features and produce a high-resolution output, which is then multiplied with the global memory feature to predict the mask. The proposed framework significantly improves segmentation performance and demonstrates the potential to be employed for teeth numbering. Rigorously evaluated on the publicly available UFBA-UESC dental image dataset, our experimental results demonstrate that the proposed model significantly outperforms existing state-of-the-art techniques in terms of segmentation performance and robustness.

This paper is organized as follows: Section 2 provides the background and related work. Section 3 offers a detailed description of the network and dataset. Section 3.4 is dedicated to the experimental setup, and then Section 4 presents the results and discussion. Finally, Section 5 concludes the paper and provides the future directions.

## 2. Related Work

There have been numerous attempts by researchers to develop teeth segmentation techniques that can be applied to various types of radiographic images, such as panoramic, periodical, and bitewing imaging. Silva et al. [30] presented a comparison of various segmentation techniques applied in dental imaging, categorizing solutions into five groups and evaluating them based on accuracy, specificity, precision, recall, and F1-score. However, all these techniques struggled to fully segment the teeth due to the presence of the bone structure inside the buccal cavity.

Classic image processing techniques have been utilized to address these challenges. For instance, to counteract the problem of low contrast, Lin et al. [31,32] first enhanced the image to distinguish between teeth and gums before applying edge extraction methods for segmentation. In a similar vein, Chandran et al. [33] improved the quality of dental images by applying CLAHE, followed by the Otsu threshold method for teeth segmentation. Level set methods have been utilized by studies [34,35] to enhance the root contrast, thus improving segmentation. Horizontal and vertical integral projection methods have also been deployed, although their performance was not satisfactory [36,37].

Recently, deep learning (DL)-based techniques have garnered attention across various industrial applications due to their impressive performance [38,39,40]. These applications span object classification [41], segmentation [42,43,44], counting [45], medical image enhancement [46,47], and object detection [48]. Specifically, in tasks such as object detection and segmentation, DL-based methods have revolutionized the field [49]. As a result, several DL-based techniques have been employed to enhance teeth segmentation in dental panoramic X-ray images. While some studies have focused solely on the semantic segmentation of teeth, limiting the level of detail for further processing steps in most automatic dental analyses [30,50,51], others have identified teeth alongside segmentation, providing more information for automatic analysis. However, these instance segmentation techniques, which typically consist of two stages, ROI/fuzzy boundary detection and teeth segmentation, increase the complexity and are more prone to errors due to their cascading nature. The errors from the first stage can propagate to the second, limiting the performance of these methods. Additionally, the information obtained from instance segmentation may not be sufficient for a comprehensive teeth analysis, as apart from intra-teeth segmentation, it is crucial to accurately segment the teeth from other oral tissues.

For instance, Jader et al. [11] employed the mask-region-based convolutional neural network (Mask-R-CNN) for instance segmentation. Their method, evaluated on a diverse set of 1500 images, achieved an accuracy of 98%, an F1-score of 88%, a precision of 94%, a recall of 84%, and a specificity of 99% over 1224 unseen images, considerably outperforming 10 unsupervised methods. However, the method was limited to teeth detection and did not account for other issues such as dentures and areas with missing teeth. Similarly, Zhang et al. [52] utilized deep-learning-based methods to detect and classify teeth, merging the Faster R-CNN and region-based fully convolutional networks (R-FCN) to identify common patient issues such as tooth loss, decay, and fillings. Similarly, Koch et al. [50] employed the U-Net architecture in conjunction with an FCN for semantic segmentation of dental panoramic radiographs and explored ways to improve segmentation performance, such as network ensembling, test-time augmentation, bootstrapping of low-quality annotations, and data symmetry exploitation. In their study, Lee et al. [53] utilized data augmentation techniques such as rotation, flipping, Gaussian blur, and shear transformation to generate 1024 training samples from 30 radiographs. They implemented a fully deep learning method using the Mask R-CNN model through a fine-tuning process to detect and localize tooth structures, achieving an F1 score of 0.875 and a mean IoU of 0.877. Muresan et al. [54] proposed a novel approach for automatic teeth detection and dental problem classification using panoramic X-Ray images. They utilized a CNN model trained on their collected data and employed image pre-processing techniques to refine segmentation, resulting in an F1 score of 0.93.

Building upon previous efforts, Zhao et al. [55] introduced a dual-stage scheme, TSASNet, to address specific issues like fuzzy tooth boundaries resulting from poor contrast and intensity distribution in dental panoramic X-rays. The method, tested on a dataset of 1500 radiographs, achieved an impressive accuracy of 96.94%, a Dice score of 92.72%, and a recall of 93.77%. Kong et al. [56] have made a substantial contribution to the scientific community by introducing a publicly available dataset that includes 2602 panoramic dental X-ray images. Each image in the dataset is paired with expertly annotated segmentation masks, thereby significantly enriching this resource. Harnessing the power of this dataset, they engineered a proficient encoder–decoder network named EED-Net. This network is specifically designed for the autonomous segmentation of the maxillofacial region, demonstrating their innovative application of the dataset. Arora et al. [57] recently introduced a multimodal encoder-based architecture, designed to extract a variety of features from panoramic radiographs. These extracted features were subsequently processed through a deconvolutional block to generate the final segmentation mask. By achieving precision and recall rates of 95.01% and 94.06%, respectively, this approach outperformed other leading methods.

In a different approach, Almalki et al. [58] utilized self-supervised learning methods, such as SimMIM and UM-MAE, to boost model efficiency in comprehending a limited number of available dental radiographs. Their SimMIM method yielded the highest performance, achieving 90.4% and 88.9% in detecting teeth and dental restorations and instance segmentation, respectively. This outperformed the random initialization baseline by an average precision increase of 13.4 and 12.8. However, the method’s requirement for extensive parameter fine-tuning creates challenges in achieving optimal results. Recently, Hou et al. [59] proposed the Teeth U-Net model. This model combines a Squeeze-Excitation Module in both the encoder and decoder, supplemented by a dense skip connection, in an attempt to bridge the semantic gap. The model also includes a Multi-scale Aggregation attention Block (MAB) in the bottleneck layer to effectively extract teeth shape features and adaptively fuse multi-scale features. To incorporate dental feature information from a broader field of view, they devised a Dilated Hybrid self-Attentive Block (DHAB) at the bottleneck layer. This block is designed to suppress irrelevant background region information without increasing the network parameters. Although the study showcased competitive performance on a private dataset, it has not yet been evaluated on publicly available datasets.

Table 1 summarizes the strides made by the aforementioned studies towards accurately segmenting teeth in panoramic radiographs.

## 3. Materials and Methods

### 3.1. Datasets

Silva et al. [30] released the UFBA-UESC Dental Images Dataset, which initially contained 1500 panoramic images along with semantic segmentation of teeth. Jader et al. [11] later introduced instance segmentation, leading to the creation of the UFBA-UESC Dental Images Deep dataset. This new dataset comprises a total of 276 images designated for training and validation. Further development by Silva et al. [7] involved the addition of tooth number information, resulting in a cumulative dataset of 543 images, inclusive of those from the UFBA-UESC Deep dataset. Named the DNS (Detection, Numbering, and Segmentation) Panoramic Images, this dataset comes equipped with binary masks and annotations in the COCO format. Detailed information about the UFBA-UESC Dental Images Dataset’s characteristics is depicted in Table 2.

For our study, we adjusted the annotations of the DNS Panoramic Images dataset for panoptic segmentation. We achieved this by merging the provided semantic and instance labels and converting them into TFRecords for our experiment. This dataset served for both training and validation, with 500 images set aside for the training set and 43 images allocated for validation. Testing images were sourced from the original UFBA-UESC Dental Images dataset.

Our research utilized the UFBA-UESC Dental Images Deep dataset [7]. This dataset is accessible through a reasonable request made to the corresponding author (https://github.com/IvisionLab/dns-panoramic-images-v2 (accessed on 2 May 2023)). Table 3 provides comprehensive details regarding the dataset, such as the presence of thirty-two teeth, restorations, and appliances, as well as the total number of images used for numbering, instance segmentation, and SS. We excluded images from categories 5 and 6 due to the presence of implants and deciduous teeth.

### 3.2. Network Architecture

**Figure 2:**
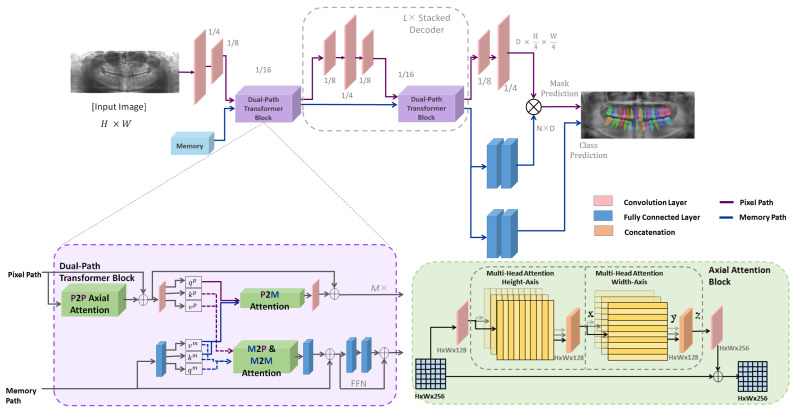
The structure of the proposed framework. An image and global memory are input into a dual-path transformer, which directly generates a collection of masks and classes (excluding residual connections). A dual-path transformer block is designed with all four types of attention (M2P, M2M, P2M, and P2P) between the two paths. On the right bottom side, the structure of the axial-attention block is illustrated. The axial attention mechanism decomposes the 2D attention into two 1D attentions; one applied along the height axis of the image, and the other applied along the width axis. By doing so, it significantly reduces the complexity from quadratic to linear, which makes it more computationally efficient.

The proposed model employs a network architecture comprised of three primary components: a Transformer block, a stacked decoder, and output heads. This end-to-end instance segmentation model predicts masks and their corresponding classes directly. In this study, we utilize Mask Transformer-Based Networks (M-TransNet) integrated with PQ Loss [62]. These networks function as instance segmentation models inspired by panoptic segmentation. The M-TransNet directly predicts class-labeled masks for panoptic segmentation, with PQ-style loss employed to train the model. This section also introduces the dual-path transformer architecture and the auxiliary losses that significantly facilitate the model’s training. A complete network diagram is displayed in Figure 2.

#### 3.2.1. Architecture Formulation

The overarching goal of panoptic segmentation is to segment every object in an image $I\in R^{H\times W\times 3}$ and assign a class label to each mask. The ground truth for a panoptic segmentation model can be expressed as:

(1) $${\left\{y_{i}\right\}}_{i=1}^{K}={\left\{\left(m_{i},c_{i}\right)\right\}}_{i=1}^{K}$$

where *K* represents the total number of non-overlapping ground truth masks $m_{i}\in {0,1}^{H\times W}$ and $c_{i}$ denotes the class label for each $m_{i}$. The output from our proposed network should precisely mirror the ground truth, thereby predicting the mask of each object alongside the class labels.

(2) $${\left\{{\hat{y}}_{i}\right\}}_{i=1}^{N}={\left\{\left({\hat{m}}_{i},{\hat{p}}_{i}\left(c\right)\right)\right\}}_{i=1}^{N}$$

where *N* remains constant and is greater than *K*, with ${\hat{p}}_{i}\left(c\right)$ representing the probability of mask $m_{i}$ being associated with class c. The network is optimized to assign an empty class to masks where *N* exceeds *K*. The class label for each mask can be predicted by taking the argmax of class probabilities:

(3) $${\hat{c}}_{i}=\underset{c}{\arg\max}\left({\hat{p}}_{i}\left(c\right)\right)$$

Similarly, the mask-ID can be assigned to each pixel by applying argmax again:

(4) $$\begin{array}{c} \hat{z}h,w=\underset{i}{\arg\max}\left({\hat{m}}_{i},h,w\right)\;\forall h\in 1,2,\ldots ,H,\;\forall w\in 1,2,\ldots ,W \end{array}$$

Each argmax is filtered using a confidence threshold. Masks or pixels with a low confidence score are removed.

#### 3.2.2. Transformer Block

The dual-path transformer module comprises two paths: a CNN path and a memory path. The CNN path processes the input image and extracts features, while the memory path stores information about the objects and their relationships within the scene. The two paths communicate through a set of attention mechanisms, which allows the model to selectively attend to different parts of the input and memory.

The CNN path within the dual-path transformer module is a standard convolutional neural network that processes the input image and extracts features. The features are passed through a series of convolutional layers, followed by a set of axial-attention blocks that implement pixel-to-pixel (P2P) self-attention. The output of the CNN path is a feature map encoding information about the input image.

The memory path in the dual-path transformer module is a memory-augmented transformer that stores information about the objects and their relationships within the scene. The memory is initialized with a set of learned object queries, which are used to attend to the input feature map and extract object features. These object features are then stored in the memory, along with their corresponding object queries. The memory is updated at each time step using a set of memory update operations, which enable the model to reason about the relationships between different objects in the scene.

The two paths in the dual-path transformer module communicate through a set of attention mechanisms. These mechanisms enable the model to selectively attend to different parts of the input and memory, allowing the model to reason about the relationships between different parts of the image and memory.

By using a dual-path transformer module, the architecture effectively combines the strengths of both CNNs and transformers for panoptic segmentation. The CNN path extracts rich visual features from the input image, while the memory path reasons about the relationships between different objects in the scene. The attention mechanisms facilitate communication between the two paths, allowing the model to selectively attend to the most relevant information for the task at hand.

#### 3.2.3. Attention Mechanisms

The attention module in the network is a key component of the memory-augmented transformer. It allows the model to selectively focus on different parts of the input image and memory, based on their relevance to the task at hand. Specifically, the attention module computes a set of attention weights for each position in the input feature map or memory, based on its similarity to other positions. These weights are then used to compute a weighted sum of the feature map or memory, which is passed through a feedforward network to produce the final output.

The dual-path transformer block employs four types of attention to facilitate communication between the CNN path and the memory path:

- Memory-to-pixel (M2P) attention: This type allows the model to attend to the memory from the pixel path. It computes attention weights for each position in the input feature map, based on its similarity to the memory.
- Memory-to-memory (M2M) self-attention: This type allows the model to attend to the memory from the memory path. It computes attention weights for each position in the memory, based on its similarity to other positions in the memory.
- Pixel-to-memory (P2M) feedback attention: This type allows the model to attend to the memory from the pixel path, but also allows the memory to attend back to the pixel path. It computes attention weights for each position in the memory, based on its similarity to the input feature map.
- Pixel-to-pixel (P2P) self-attention: This type allows the model to attend to the input feature map from the pixel path. It computes attention weights for each position in the input feature map, based on its similarity to other positions in the input feature map. In the network, P2P self-attention is implemented as axial-attention blocks, which are more efficient than global 2D attention on high-resolution feature maps.

#### 3.2.4. Decoder Block and Output Heads

The decoder block is stacked *L* times, iterating through output strides (4, 8, and 16 [63,64]) multiple times at each decoding resolution. It merges features by performing bilinear resizing, simple summation, and applying either convolutional blocks or transformer blocks before moving to the next resolution. While it shares similarities with feature pyramid networks [65,66] designed for pyramidal anchor predictions [67], the purpose of our decoder block is solely to aggregate multi-scale features without directly using intermediate pyramidal features for prediction.

The output heads are designed to make predictions from the processed features. Following the stacked decoder, two fully connected layers (2FC) and a softmax function predict mask classes using the memory feature of length *N*. For mask prediction, the decoder block is followed by 2FC to obtain a memory path mask feature (*f*). The decoder output at stride 4 passes through two convolution layers (2Conv) to generate the normalized pixel path feature (*g*). The predicted mask is then obtained from the multiplication of *f* and *g*, where $f\in R^{N\times D}$ and $g\in R^{D\times \frac{H}{4}\times \frac{W}{4}}$.

#### 3.2.5. Combining Outputs for Panoptic Segmentation

The network directly predicts class-labeled masks using a mask transformer, which outputs a set of instance masks and a semantic mask. The instance masks represent the pixels belonging to each object instance in the scene, while the semantic mask represents the pixels belonging to each semantic class.

To obtain the final panoptic segmentation, the instance masks and the semantic mask are combined using a post-processing step. Specifically, the instance masks are first grouped into object instances using a clustering algorithm, such as mean-shift or DBSCAN. The resulting object instances are then assigned a unique instance ID, used to distinguish them from other object instances in the radiographs.

Next, the semantic mask is merged with the instance masks to obtain the final panoptic segmentation of teeth. This is achieved by assigning each pixel in the semantic mask to the object instance to which it belongs, based on the instance ID of the corresponding pixel in the instance masks.

### 3.3. Loss Function

For training, we used a main loss function and auxiliary losses. Panoptic segmentation comprises two main tasks: segmentation and recognition. Therefore, an optimal loss function should check the quality of both. Our main loss function is a product of recognition quality (RQ) and segmentation quality (SQ). The loss function basically maximises a similarity metric over matched masks. One-to-one bipartite matching between the predicted and ground truth masks is performed first, followed by the computation of the similarity metric that can be given as:

(5) $$\mathrm{sim}\left(y_{i},{\hat{y}}_{j}\right)={\hat{p}}_{j}\left(c_{i}\right)\times \mathrm{Dice}\left(m_{i},{\hat{m}}_{j}\right)$$

where sim(·,·) is the mask similarity metric between class-labelled ground truth mask $y_{i}=\left(m_{i},c_{i}\right)$ and predicted mask ${\hat{y}}_{j}=\left({\hat{m}}_{j},{\hat{p}}_{j}\left(c\right)\right)$. The similarity metric ranges between 0 and 1. The value will be 0 when the class is wrong or the masks do not overlap, while it will be 1 when both the classes and masks match precisely. For mask matching, each predicted mask is matched with the ground truth until maximum total similarity is achieved using one-to-one bipartite matching, which is given as:

(6) $$\hat{\sigma }=\underset{\sigma \in S_{N}}{\arg\max}\sum_{i=1}^{K}\mathrm{sim}\left(y_{i},{\hat{y}}_{\sigma (i)}\right)$$

where ${\left\{{\hat{y}}_{i}\right\}}_{i=1}^{N}$ and ${\left\{y_{i}\right\}}_{i=1}^{K}$ are the prediction and ground truth sets, respectively, and $\sigma \in S_{N}$ is the permutation of *N* elements that best assigns the predictions to obtain maximum similarity. Considering the similarity metric and the mask-matching process, the loss function can be given as:

(7) $$\begin{array}{cc} L_{\mathrm{PQ}}^{\mathrm{pos}} & =\sum_{i=1}^{K}\underset{\mathrm{weight}\;}{\underset{︸}{{\hat{p}}_{\hat{\sigma }\left(i\right)}\left(c_{i}\right)}}\cdot \underset{\mathrm{Dice}\;\mathrm{loss}\;}{\underset{︸}{\left[-\mathrm{Dice}\left(m_{i},{\hat{m}}_{\hat{\sigma }\left(i\right)}\right)\right]}}\\ \; & +\sum_{i=1}^{K}\underset{\mathrm{weight}\;}{\underset{︸}{\mathrm{Dice}\left(m_{i},{\hat{m}}_{\hat{\sigma }\left(i\right)}\right)}}\cdot \underset{\mathrm{Cross}-\mathrm{entropy}\;\mathrm{loss}\;}{\underset{︸}{\left[-\log{\hat{p}}_{\hat{\sigma }\left(i\right)}\left(c_{i}\right)\right]}} \end{array}$$

Intuitively, we optimize the dice loss weighed by class correctness and the cross-entropy loss weighted by mask correctness as we want both class and mask to be correct at the same time. Apart from $L_{\mathrm{PQ}}^{\mathrm{pos}}$ for positive masks, we define a cross-entropy term $L_{\mathrm{PQ}}^{\mathrm{neg}}$ for negative (unmatched) masks:

(8) $$L_{\mathrm{PQ}}^{\mathrm{neg}}=\sum_{i=K+1}^{N}\left[-\log{\hat{p}}_{\hat{\sigma }\left(i\right)}\left(⌀\right)\right]$$

This term trains the model to predict ⌀ for negative masks. We balance the two terms by α as a common practice to weight positive and negative samples:

(9) $$L_{\mathrm{PQ}}=\alpha L_{\mathrm{PQ}}^{\mathrm{pos}\;}+\left(1-\alpha \right)L_{\mathrm{PQ}}^{\mathrm{neg}}$$

where $L_{\mathrm{PQ}}$ denotes our final PQ-style loss. In addition to the PQ-style loss, we also use three other losses: (1) Instance discrimination, used while learning feature maps. This loss helps cluster decoder features into instances. (2) Mask ID cross entropy, helps classify each pixel into *N* masks. (3) Semantic segmentation loss, helps in separating the final mask features.

### 3.4. Experimental Setup

#### 3.4.1. Training

All experiments were conducted using the UFBA-UESC dataset. The proposed network was implemented with the Tensorflow framework. Training was performed on an NVIDIA RTX Titan GPU for 500 epochs.

#### 3.4.2. Evaluation Parameters

The following evaluation metrics were used to compare our results with state-of-the-art segmentation models, where the *F*1 *score* was mainly used as a reference since it can give a better estimation of overall performance.

(10) $$Accuracy=\frac{TP+TN}{TP+FN+TN+FP}$$

(11) $$Specificity=\frac{TN}{TN+FP}$$

(12) $$Precision=\frac{TP}{TP+FP}$$

(13) $$Recall=\frac{TP}{TP+FN}$$

(14) $$F1\;Score=\frac{2\times Precision\times Recall}{Precision+Recall}$$

## 4. Results

**Figure 3:**
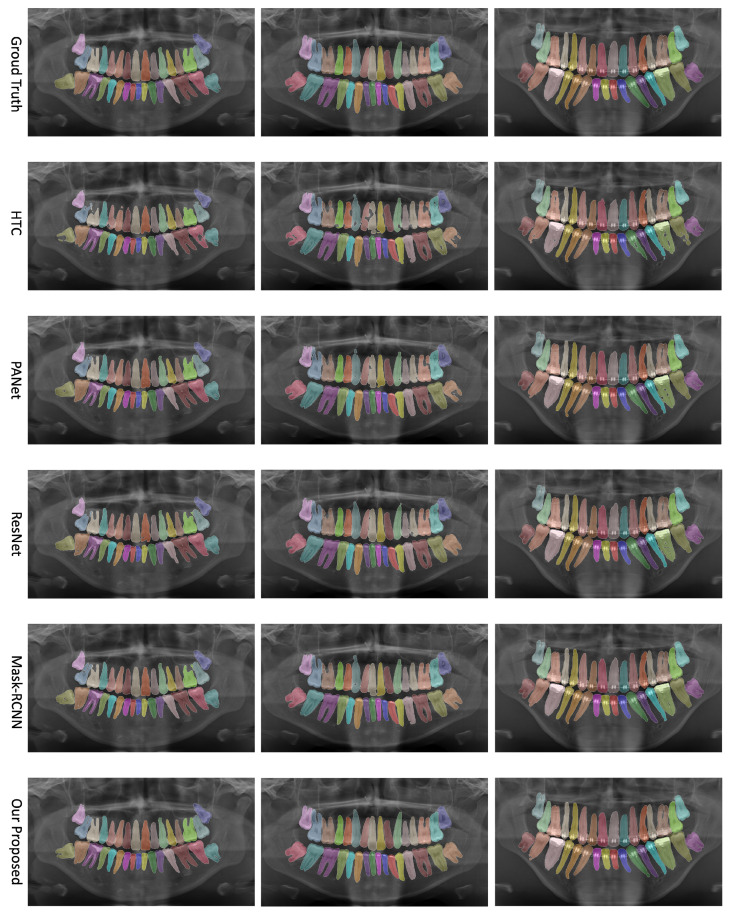
Comparison of teeth instance segmentation results for various networks—PANet, HTC, Mask R-CNN, ResNet, and our proposed approach—alongside the ground truth.

We evaluate the performance of our proposed network on the UFBA-UESC Dental Images dataset. Our analysis includes both quantitative and qualitative assessments, comparing our results to those of other state-of-the-art techniques. This section provides a comprehensive discussion of our evaluation results. Figure 3 presents a visual comparison of instance segmentation results produced by various networks (i.e., PANet, HTC, Mask R-CNN, ResNet, and our approach) alongside the ground truth.

### 4.1. Ablation Study

We also performed an ablation study to understand the contribution of different components of our network better. This study focused on a subset of the dataset and examined changes in the *F*1*-score*, *Precision*, and *Recall* as we removed different components. We have summarized the results in Table 4.

The ablation study provides valuable insights into the performance impact of each network component. For instance, the transformer block greatly enhances the performance by enabling efficient bi-directional communication between the pixel path CNN and memory path. Similarly, the stacked decoder, which plays a critical role in aggregating multi-scale features, helps to improve the accuracy of the segmentation output. The output heads are responsible for predicting mask classes and have a direct impact on the network’s performance. The pixel-to-memory (P2M) feedback attention, a component of the transformer block, allows for the selective aggregation of information from memory, enabling the model to capture context-aware features, thus leading to improved teeth segmentation. Both the memory-to-pixel (M2P) and memory-to-memory (M2M) self-attention mechanisms demonstrated their significance by capturing long-range dependencies within the memory path and providing global context information.

### 4.2. Qualitative Analysis

**Figure 4:**
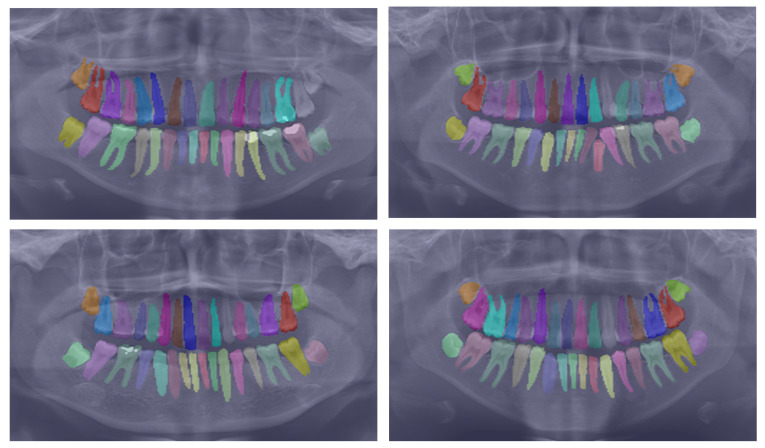
Showcasing the best panoptic segmentation results that encompass both the semantic segmentation of the background class and the instance segmentation of the teeth classes.

**Figure 5:**
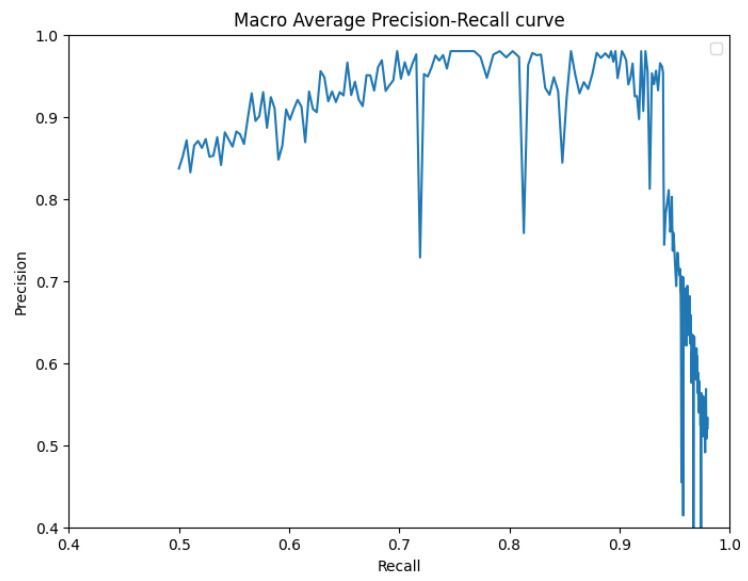
Precision–recall curve.

To further substantiate our comparison, we visualized the results from our proposed model. Figure 3 displays the instance segmentation results of various networks compared to the ground truth. Our method demonstrates closer alignment with the ground truth, indicating better performance in teeth instance segmentation tasks compared to the other methods. Notably, our proposed network maintains a consistent performance across all teeth, unlike the other networks. The synergistic benefits of the two tasks, SS and affinity pyramid, primarily drive the improvement in instance segmentation performance. Figure 4 depicts the results of panoptic segmentation with the background class (semantic segmentation) and tooth classes (instance segmentation). Figure 5 presents the precision–recall curve, which is the average of precision and recall for all classes. Panoptic segmentation improves the Dice score by also considering the surrounding tissues of teeth; thus, the loss also takes into account the background segmentation to yield better results.

### 4.3. Comparison with State-of-the-Art Models

Next, we compared our model with state-of-the-art approaches in the context of instance segmentation and SS. Table 5 demonstrates that our proposed framework outperforms all previously proposed methods. Mask R-CNN [30] and the TSAS-Net [55] have both been utilized for teeth segmentation, while PANet [7] has achieved state-of-the-art results. However, our approach surpasses these existing methods by capturing hidden patterns more effectively and providing more accurate segmentation of human teeth, even in challenging scenarios like overlapping teeth masks.

We further evaluated the performance of our proposed method in comparison to previously published studies related to teeth segmentation in panoramic radiographs. Table 6 summarizes the results, which underscore the remarkable performance of our proposed scheme. Given the impressive performance of our framework, as substantiated by our experimental results, we assert that our proposal has established a new state of the art in teeth segmentation.

### 4.4. Limitations

Our proposed method seeks to achieve instance segmentation of teeth in panoramic radiographs by leveraging an end-to-end model specifically designed for panoptic segmentation. This innovative approach unifies semantic and instance segmentation tasks, introducing a dual-path architecture that adds a global memory path to the conventional CNN path. This unique setup facilitates direct communication across all CNN layers. The architecture explicitly crafted for panoptic segmentation leverages novel objectives, providing equal treatment to both semantic regions and instance objects. As a result, the proposed scheme significantly enhances the instance segmentation performance of teeth in panoramic radiographs. Despite these notable advancements, the proposed approach does introduce certain challenges. One key limitation lies in its additional computational complexity, which may impede real-time clinical applications. Furthermore, our evaluation of the proposed method relies solely on a single dataset. This limited scope constrains a comprehensive assessment of the scheme’s generalization capabilities, restricting its potential for a more universally applicable evaluation.

## 5. Conclusions and Future Directions

We have applied a panoptic segmentation strategy to conduct instance segmentation of teeth in panoramic radiographs. Our approach uniquely intertwines the instance segmentation of teeth with the semantic segmentation of the background, enhancing intra-teeth classification and enabling our architecture to accurately distinguish teeth from oral tissue. Our method incorporates an end-to-end deep learning model, which leverages a mask transformer to predict class-labelled masks directly. This is accomplished via a dual-path architecture that introduces an additional global memory path alongside the CNN path, thus enabling direct communication with any CNN layer. We trained our model utilizing a panoptic-quality-inspired loss through bipartite matching. As a result, our proposed framework attains a significantly improved segmentation performance, which also proves beneficial for teeth numbering. The proposed method underwent rigorous evaluation on the publicly accessible UFBA-UESC Dental Image dataset. The experimental results validate that our proposed model outstrips existing state-of-the-art techniques in terms of segmentation performance and robustness.

Looking ahead, our future work aims to further enhance the dual-path-based mask transformer architecture. A key focus will be enabling the numbering of teeth in panoramic radiographs, a crucial factor for accurate tooth identification that significantly aids in diagnosis, treatment planning, and effective communication among dental professionals.

## Figures and Tables

**Table 1:** Summary of previously published methods for teeth segmentation in panoramic radiographs.

Authors, Year	Technique	Contribution/Advantages	Limitations
Jader et al. [11], 2018	Instance segmentation for panoramic X-ray images	Introduced a new instance segmentation technique for teeth segmentation with promising results.	Struggles with overlapping and adjacent teeth.
Zhang et al. [52], 2018	Label tree with cascade network structure for teeth recognition	Improved teeth recognition using a novel label tree and cascade network structure.	Inefficient with teeth suffering from severe pathologies.
Koch et al. [50], 2019	U-Nets for dental panoramic radiographs segmentation	Developed an accurate tooth segmentation technique based on U-Nets. Demonstrated improved performance.	Difficulty in segmenting teeth with complex structures or deformities.
Lee et al. [53], 2020	Deep convolutional neural network for tooth segmentation automation	Employed a deep convolutional neural network for automated tooth segmentation. Enhanced both efficiency and accuracy.	Limitations when dealing with noisy or poor-quality images.
Muresan et al. [54], 2020	Deep learning and image processing techniques for teeth detection and dental problem classification	Introduced a novel approach using deep learning and image processing techniques for teeth detection and dental problem classification.	Struggles with dental problems underrepresented in the training data.
Zhao et al. [55], 2020	TSASNet: Two-Stage Attention Segmentation Network for tooth segmentation	Developed TSASNet, a Two-Stage Attention Segmentation Network for tooth segmentation, showing enhanced results.	Inefficient with teeth of unusual shapes or sizes.
Kong et al. [56], 2020	Efficient encoder–decoder network for automated maxillofacial segmentation	Proposed an automated segmentation method for maxillofacial regions in dental X-ray images. Showed improved efficiency and accuracy.	Difficulty with radiographs containing artifacts or of poor quality.
Shubhangi et al. [60], 2022	CNNs combined with classical image processing methods	Performed teeth segmentation and numbering using a histogram-based plurality vote process.	Computationally expensive, posing challenges for real-time applications.
Arora et al. [57], 2023	Multimodal encoder-based architecture	Achieved superior teeth segmentation performance.	Limited to semantic segmentation.
Datta et al. [61], 2023	Combination of neutrosophic logic and a fuzzy c-means algorithm	Demonstrated competitive performance.	Relies on conventional image processing techniques, which might lack robustness.
Almalki et al. [58], 2023	Self-supervised learning methods (i.e., SimMIM and UM-MAE) for dental panoramic radiographs	SimMIM, a masking-based method, outperformed UM-MAE and supervised and random initialization methods for teeth and dental restoration detection and instance segmentation.	Parameter fine-tuning, including mask ratio and pre-training epochs, substantially influence segmentation performance.
Hou et al. [59], 2023	UNet with dense skip connection and attention units	Used dense skip connections and attention units to handle the irregular shape of teeth. Introduced Multi-scale Aggregation Attention Block (MAB) and Dilated Hybrid self-Attentive Block (DHAB) at the bottleneck layer.	Lacks performance analysis on public datasets, making a fair comparison challenging.

**Table 2:** UFBA-UESC Dental Images Dataset characteristics. Note that ✓ and – represent the presence and absence of category, respectively.

Category	Restoration	Appliance	Teeth Numbers	Image Numbers
1	✓	✓	32	73
2	✓	–	32	220
3	–	✓	32	45
4	–	–	32	140
5	–	–	18	120
6	–	–	37	170
7	✓	✓	27	115
8	✓	–	29	457
9	–	✓	28	45
10	–	–	28	115
Total	–	–	–	1500

**Table 3:** Dataset characteristics used in this work. Note that ✓ and – represent the presence and absence of the corresponding category, respectively.

Category	32 Teeth	Restoration	Appliance	Number and Instance Segmentation	Segmentation
1	✓	✓	✓	23	57
2	✓	✓	–	174	80
3	✓	–	✓	42	11
4	✓	–	–	92	68
7	–	✓	✓	36	87
8	–	✓	–	128	355
9	–	–	✓	14	33
10	–	–	–	34	87
Total	–	–	–	543	778

**Table 4:** Ablation study results.

Component Removed	Accuracy	F1-Score	Precision	Recall
None (Full model)	97.25	93.47	95.13	93.92
Transformer Block	95.68	91.34	92.81	90.53
Stacked Decoder	95.04	90.12	91.57	88.84
Output Heads	94.12	88.90	90.36	87.66
Pixel-to-Memory	95.32	90.77	92.20	89.48
Memory-to-Pixel	95.56	91.22	92.62	89.97

**Table 5:** Comparison with state-of-the-art methods, the best results are indicated in bold.

Method	Accuracy	Specificity	Precision	Recall	F1-Score	mAvP	AvP50	AvP75
Mask R-CNN [30]	92.08	96.12	83.73	76.19	79.44	66.4 ± 0.7	96.9 ± 0.2	85.1 ± 1.0
TSAS-Net [55]	96.94	97.81	94.97	93.77	92.72	70.9 ± 0.1	97.7 ± 0.1	89.7 ± 0.5
PANet [7]	96.7	98.7	94.4	89.1	91.6	71.3 ± 0.3	97.5 ± 0.3	88.0 ± 0.2
HTC	96	98.5	93.7	85.9	89.6	63.7 ± 1.4	97.0 ± 0.0	82.2 ± 2.0
UNet	96.04	97.68	89.89	90.18	89.33	67.0 ± 0.5	96.3 ± 0.2	87.7 ± 0.9
Ours	**97.25**	**97.65**	**95.13**	**93.92**	**93.47**	**71.5 ± 0.2**	**98.1 ± 0.4**	**89.2 ± 0.1**

**Table 6:** Comparison with previously published studies, the best results are indicated in bold.

Method	Accuracy	Specificity	Precision	Recall	F1-Score
Wirtz et al. [51]	–	–	79	82.7	80.3
Lee et al. [53]	–	–	85.8	89.3	87.5
Arora et al. [57]	96.06	**99.92**	95.01	93.06	91.6
Fatima et al. [68]	–	–	86	87	84
Karaoglu et al. [69]	–	–	93.33	93.33	93.16
Proposed Method	**97.25**	97.65	**95.13**	**93.92**	**93.47**

## Data Availability

The data used in this study are openly available in [7] via https://github.com/IvisionLab/dns-panoramic-images-v2 (accessed on 2 May 2023).
